# Electromagnetic fields regulate calcium-mediated cell fate of stem cells: osteogenesis, chondrogenesis and apoptosis

**DOI:** 10.1186/s13287-023-03303-w

**Published:** 2023-05-16

**Authors:** Tian Ma, Qing Ding, Chaoxu Liu, Hua Wu

**Affiliations:** Tongji Hospital, Tongji Medical College, Huazhong University of Science and Technology, Wuhan, 430030 Hubei China

**Keywords:** Electromagnetic fields, Calcium ion, Calcium oscillations, Stem cells, Tumor stem cells, Biosafety

## Abstract

Electromagnetic fields (EMF) are increasing in popularity as a safe and non-invasive therapy. On the one hand, it is widely acknowledged that EMF can regulate the proliferation and differentiation of stem cells, promoting the undifferentiated cells capable of osteogenesis, angiogenesis, and chondroblast differentiation to achieve bone repair purpose. On the other hand, EMF can inhibit tumor stem cells proliferation and promote apoptosis to suppress tumor growth. As an essential second messenger, intracellular calcium plays a role in regulating cell cycle, such as proliferation, differentiation and apoptosis. There is increasing evidence that the modulation of intracellular calcium ion by EMF leads to differential outcomes in different stem cells. This review summarizes the regulation of channels, transporters, and ion pumps by EMF-induced calcium oscillations. It furtherly discusses the role of molecules and pathways activated by EMF-dependent calcium oscillations in promoting bone and cartilage repair and inhibiting tumor stem cells growth.

## Introduction

**Fig. 1:**
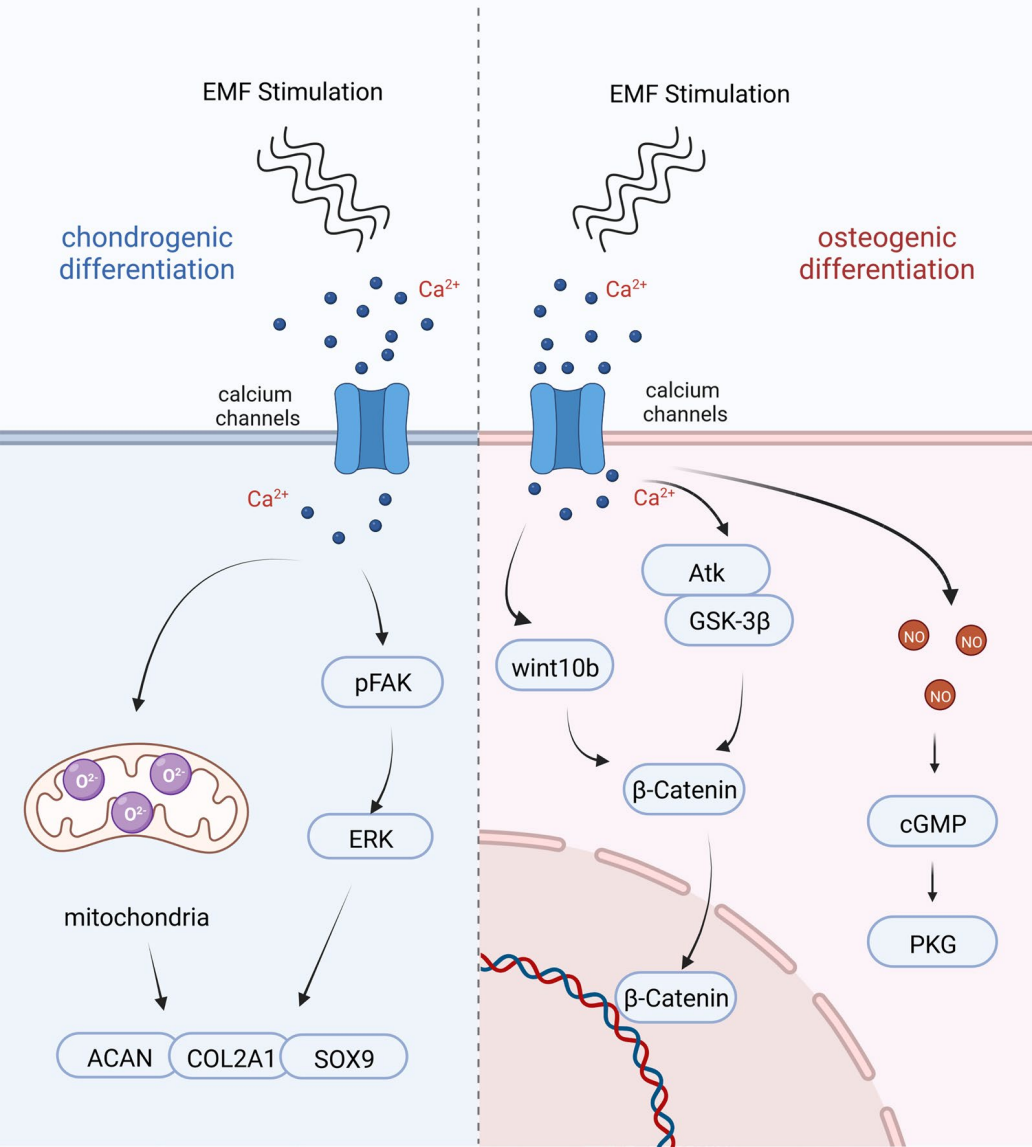
Cascades of EMF-induced calcium ion. Electromagnetic fields stimulation induces calcium oscillations in stem cells, and activation of calcium ion after endocytosis can activate multiple downstream signaling pathways that promote stem cell proliferation, osteogenic differentiation or chondrogenic differentiation[18, 20, 40, 70, 71, 74, 79]. The figure was created with BioRender.com

Since the late nineteenth century, electromagnetic wave has been proved to exist in the physical field. With the advancement of EMF researches, Reiter R, Persinger MA, Frey AH and others have summarized the biological effects and applications of electromagnetic fields in the late twentieth century [1–3]. On this basis, Electromagnetic fields therapy has gradually been accepted and widely valued. Electromagnetic field has been active in clinical treatment and research applications as a versatile therapeutic expert for nearly 50 years, such as bone repair [4], treatment of osteoarthritis [5], treatment of degenerative nerve diseases [6] and tumor suppression [7], etc. The initiating mechanism for the complex bioregulatory effects of electromagnetic fields on different tissues and cells is still unclear, and this paper suggests that intracellular calcium ion may play a key role (Fig. 1).

Intracellular calcium homeostasis is closely related to cell fate, such as proliferation, differentiation, metabolism, apoptosis, etc. [8]. In most cases, calcium homeostasis is achieved through calcium channels in cell membranes, receptors and intracellular calcium dynamics [9]. When cells are subjected to external mechanical stimulation, voltage-gated ion channels in the cell membrane open and intracellular calcium ion concentration rises [10]. The calcium ion concentration is about 100 nM in a resting-state and rises to about 1000 nM in an activated state. Hence the upregulated calcium ion activates various calcium-sensitive cascade reactions, such as calmodulin (CaM), cAMP, NOS, Ins _(1,4,5)_ P3, which regulate cell metabolism through a complex network [11].

This review focuses on EMF—which can be used as a non-invasive treatment for a wide variety of cells—and on how it induces different cell fates by regulating calcium ion. As a biomechanical stimulus, EMF is able to exert its biomagnetic effects by generating peak currents through non-thermal effects that alter the peak or spatial distribution of intracellular calcium ion concentrations through stimulation over minutes to hours [12–14]. We summarize latest therapeutic cases of extremely low frequency electromagnetic fields (ELE-EMF) and radio frequency electromagnetic fields (RF-EMF) applied to a variety of cell types, such as stem cells, osteoblasts, and tumor cells (Table 1). Interestingly, by regulating intracellular calcium ion, EMF promotes normal stem cells in the body proliferation and differentiation, but inhibits tumor stem cells proliferation and promotes apoptosis. The contrasting effects of EMF on normal and tumor stem cells are likely to be related to the abnormalities of the tumor stem cells' own calcium ion channels [15].

**Table 1:** EMF therapeutic cases

EMF type	Parameter (frequency/intensity)	Cell type	Effect	References
EMF	15 Hz/1 mT	BMSCs	EMF combined with VEGF promote osteogenesis and angiogenesis	[16]
EMF	15 Hz/0.3 mT	BMSCs	EMF combined with PCL/nHA scaffold accelerate intervertebral fusion	[17]
EMF	45 Hz/1 m T, 8 h/day	Saos-2 osteoblast cell line	EMF combined with Fe_3_o_4_ nanoparticles promote repair of rat calvarial defect	[18]
EMF	31.4 µT, 1 h/day	ADSCs	EMF combined with PCL/CMC scaffold promote osteogenesis	[19]
PEMF	1 mT, 10 min	MSCs	EMF promote chondrogenic differentiation	[20]
AM RF EMF	27.12 MHz, 1 h**/**day	Breast cancer cells (tumor stem cells)	EMF inhibit breast cancer brain metastasis	[21]
AM RF EMF	27.12 MHz	Hepatocellular carcinoma (tumor stem cells)	EMF inhibit hepatocellular carcinoma proliferation and metabolism	[22]
EMF	1 Hz/100 mT	MC4-L2 breast cancer cells	EMF lead to calcium ion overload and ROS increased, resulting in necroptosis	[23]
EMF	50 Hz/4.5 mT	786-O cells	ELF-EMF induce G0/G1 arrest and apoptosis in cells lines	[24]

A. EMF: Electromagnetic fields. PEMF: Pulsed electromagnetic fields. AM RF EMF: Athermal radiofrequency electromagnetic fields. ELF-EMF: Extremely low frequency electromagnetic fields. B. Parameter: The unit of frequency is Hertz. The unit of intensity is Tesla. C. BMSCs: Bone mesenchymal stem cells. Saos-2 osteoblast cell line: Osteosarcoma osteoblast cell line. ADSCs: Adipose Derived Stem Cells. MSCs: Mesenchymal stem cells. 786-O cells: Human renal clear cell Carcinoma. D. PCL: Polycaprolactone. CMC: Carboxymethyl cellulose. HA: Hydroxyapatite

## EMF

EMF as a therapeutic versatile has been carried out in a large number of in vivo, in vitro and clinical trials [4, 25–27]. After conducting a large number of EMF-related in vitro experiments, our group combined EMF, stem cells, and bone tissue engineering into an organic whole, and we still achieved the repair of bone defects in a variety of animals in a more complex in vivo physiological environment compared to the relatively single, controlled environment of in vitro experiments [16, 17, 28]. To be able to better simulate the in vivo experimental environment, Sundelacruz [29] constructed a three-dimensional (3D) in vitro tissue model of trauma, in which the mineralization of osteoblasts was enhanced by EMF stimulation, recreating the process of bone regeneration. Based on the current research, the therapeutic EMFs are diverse, with pulsed and sinusoidal EMFs being common depending on the EMF waveform; researchers have also used complex combinations of parameters, ranging from very low frequency EMFs that induce osteogenic or chondrogenic differentiation of stem cells [17, 20], to radio frequency EMFs that inhibit tumor proliferation [21, 22]. During the treatment, we need to control the intensity of ELE-EMF, and when high intensity stimulation is used, the cells receive more energy, which may lead to the inhibition of cell growth and metabolism [30], implying that the electromagnetic field intensity is also an important parameter in regulating EMF effects. Based on the current study, we believe that for low or extremely low frequency EMF, frequencies in the range of 0–75 Hz and intensities in the range of 0–1 mT have osteogenic or chondrogenic biological effects, while EMF with this combination of parameters are safe for adults but prohibited for children [31], and if the electromagnetic field intensity is further increased, it may be necessary to reduce the stimulation time to ensure safety [20]. For RF EMF, these two frequencies, 27.12 MHz and 835 MHz, have biological effects for the treatment of tumors [21, 32], and no adverse effects have been reported yet.

The biological mechanisms by which EMF promote cell differentiation and apoptosis are diverse. In the initial studies, researchers suggested that EMF can directly regulate intracellular proteins or transition metal ions in enzymes to affect enzyme activity and thus regulate biochemical reactions [33]. And another part of the study suggested that EMF can regulate non-coding RNAs to further activate intracellular signaling cascade pathways [34, 35]. As the study of EMF has intensified, the mechanisms of biological effects of EMF have been divided into two main categories, namely, thermal and nonthermal effects of EMF. Although these two theories differ in the initiating factors, with the former relying on heat generated by EMF and the latter being the sum of all modes of action unrelated to magneto-thermal, both focus their researches on the effects of EMF on intracellular metal ions and ion channels opening and closing [36–38]. Despite the fact that EMF parameters are characterized by diversity and complexity, several studies have pointed out that EMF can modulate intracellular calcium oscillations [22, 39, 40].

## Mechanism of electromagnetic fields-induced calcium ion oscillations

Electromagnetic fields, as a specific biomechanical stimulus, is widely recognized to alter membrane potential, activate calcium channels, increase their activity, and as a result, cause calcium oscillations [41, 42]. After receiving biomechanical stimulation, cells can alter intracellular calcium concentration through a variety of membrane-dependent pathways: (1) Activation of L-type or T-Type voltage-gated ion channels by increasing channel activity and protein expression levels [43, 44]. (2) Activation of the purinergic receptor family on the cell membrane is activated, where mechanical signaling receptors on the cell membrane are activated, causing the ATP or ADP increases, and the purinergic signal can upregulate the expression of P2Y1 receptors [45, 46]. (3) Activation of the TRP family of cation channels, TRPV4, which exhibits moderate permeability to calcium ion, is activated to alter the permeability of the cell membrane and promote the inward flow of calcium ion [47, 48]. (4) It also activates its cation channels or proteins on the cell membrane, such as the electromagnetic perceptive gene (EPG) is able to highly express a transmembrane protein that regulates the flow of calcium ion in response to electromagnetic fields stimulus [49]. Real-time confocal imaging enables the observation of a calcium peak in cells within minutes after stimulation, and when the energy of stimulation is further increased, more cells show an activated state while the amplitude of the calcium peak increases further [50, 51]. We summarize the calcium channels, receptors and transporters regulated by EMF (Table 2).

**Table 2:** EMF-induced calcium oscillations

Channel/transporter		Cell type	Changes	EMF type	Parameter (frequency/intensity)	References
TRPC1	TRPC1	MSC	Increased	PEMF	10 min, 0–3 mT	[20]
TRPC1	TRPC1	Myoblasts	Increased	PEMF	10 min, 1.5 mT	[52]
Purinergic receptor	P2X7	Mesenchymal stem cells	Increased	EMF	7.5 Hz, 15 Hz, 50 Hz,75 Hz/1 mT	[53]
T-type VGCC	Ca_v_3·2 CACNA1H	Hepatocellular carcinoma cells (tumor stem cells)	Increased	AM RF EMF	27·12 MHz	[22]
T-type VGCC	CACNA1H	Breast cancer cells (tumor stem cells)	Increased	AM RF EMF	27.12 MHz	[21]
VGCC		Hippocampus	Decreased	RF-EMF	835 MHz	[32]
L-type VGCC		neurons	Increased	PEMF	50 Hz/1 mT	[54]
T-type VGCC		B16F10 Cancer Cells	Increased	ELF-EMF	7.83 Hz	[55]
L-type VGCC						
T-type VGCC		B16-BL6, MDA-MB-231, MCF-7, and HeLa cells	Increased	EMF	25–6 Hz	[44]
T-type VGCC	Cav3.1, Cav3.2 Cav3.3	HEK293 cells	Decreased	ELF-EMF	50 Hz/0.2 mT	[56]
Ca2^+^-ATPase	SERCA2a	Cardiomyocytes	Increased	ELF-EMF	15 Hz, 50 Hz, 75 Hz and 100 Hz/2 mT	[39]

A. MSC: Mesenchymal stem cells. B16F10, B16-BL6: Mouse melanoma cell line. MDA-MB-231: Breast carcinoma cell line. MCF-7: Human breast adenocarcinoma cell line. HeLa cells: Human cervical cancer. HEK293: Humanembryonic kidney 293. B. EMF: Electromagnetic fields. PEMF: Pulsed electromagnetic fields. AM RF EMF: Athermal radiofrequency electromagnetic fields. RF-EMF: Radiofrequency electromagnetic fields. ELF-EMF: Extremely low frequency electromagnetic fields. C. Parameter: The unit of frequency is Hertz. The unit of intensity is Tesla

**Fig. 2:**
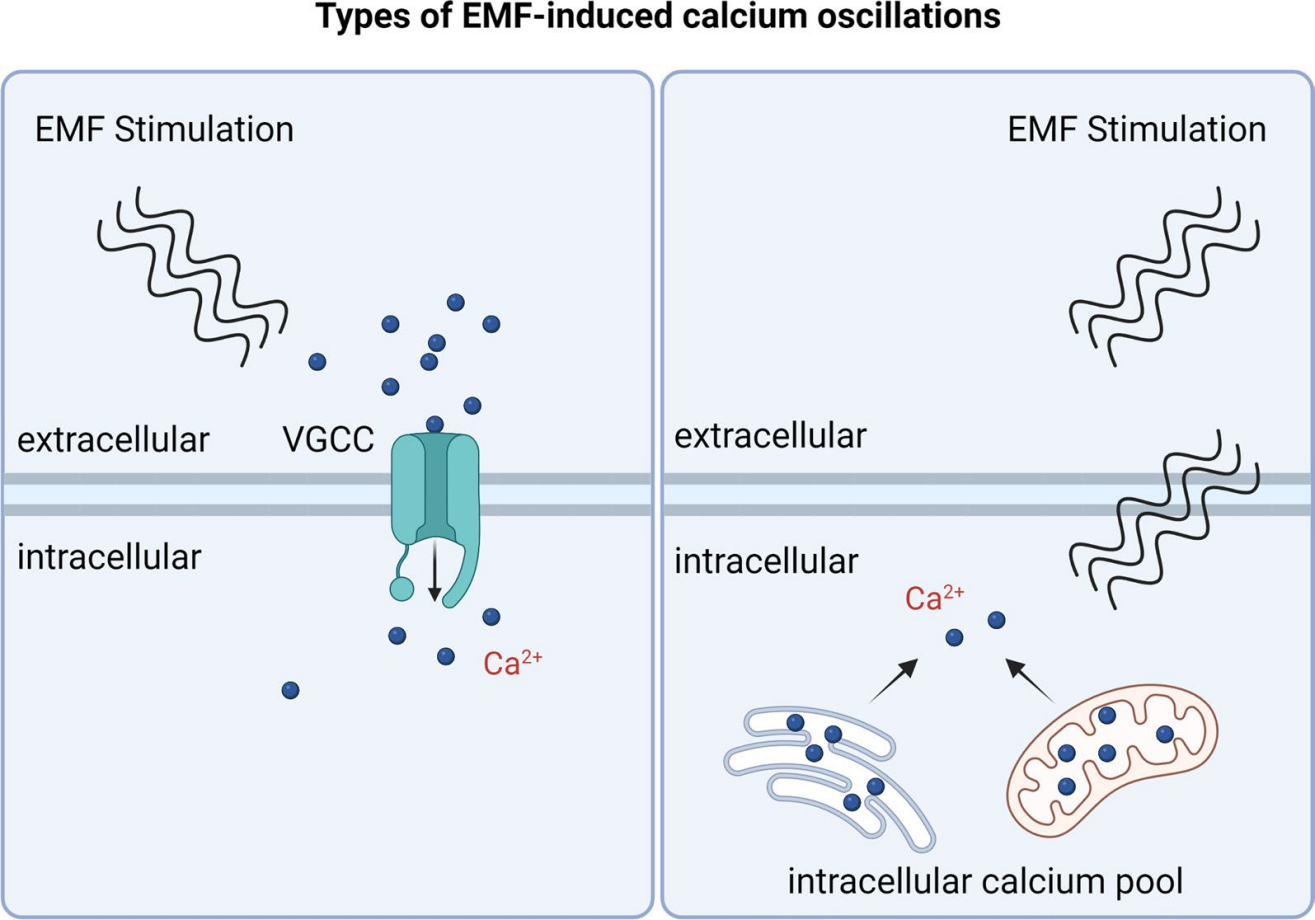
Types of EMF-induced calcium oscillations. Electromagnetic fields can activate ion channels to promote calcium inward flow and also regulate intracellular calcium pools, endoplasmic reticulum and mitochondria to cause calcium oscillations. The figure was created with BioRender.com

Calcium ion oscillations induced by EMF can be achieved by two classical calcium inward currents, the first through L-type or T-type voltage-gated channels, where the stimulation of electromagnetic fields can open ion channels located on the cell membrane, allowing the inward flow of extracellular calcium ion [55]. In the second, EMF can alter intracellular calcium ion concentrations independently of calcium ion channels on the cell membrane, causing calcium ion oscillations [57], a change that is likely due to EMF affecting intracellular calcium stores and the release of calcium ion from intracellular calcium pool [58]. The endoplasmic reticulum and mitochondria are the main storage organelles for calcium ion, and calcium ion are released from the inter-membrane space membrane into the cytoplasm during calcium oscillations, transiently or by maintaining elevated calcium ion concentrations [59–61]. After 10 min EMF stimulation of myocytes, TRPC1 channel expression is upregulated, mitochondrial respiratory capacity is enhanced, and cellular metabolism is increased [52]. Mitochondria are also able to regulate calcium ion concentration in the cellular matrix via IP3 [62], but no EMFs have been reported to be associated with this (Fig. 2).

Different calcium channels in the cell membrane are capable of responding to a variety of stimuli from the external environment, and there are various calcium channels such as mechanical force-sensitive, voltage-sensitive, and temperature-sensitive. EMF is capable of activating calcium channels through both thermal and non-thermal effects, and it has been debated which of these two effects plays the more dominant role, but the calcium oscillations induced by EMF are inextricably linked to magnetic nanoparticles [63, 64]. Among the non-thermal effects of EMF, researchers have suggested that EMF has a penetrating effect and is able to exert a mechanical force stimulus on cells, while calcium channels sensitive to mechanical forces exist in the cell membrane, and these calcium channels can be activated by external mechanical forces, causing calcium ion inward flows [65]. The mechanical force of EMF depends on other magnetic ions inside or outside the cell, such as ferromagnetic nanoparticles or ferritin [63]. The administration of ferromagnetic nanoparticles from outside the cell under the action of EMF can be observed by calcium ion fluorescence with a 20% signal enhancement and an increase in the peak frequency of calcium ion oscillations, which are not significantly affected when changing the temperature of the extracellular environment [66]. Iron oxide nanoparticles are superparamagnetic and can respond to the mechanical force of the external EMF, and if iron oxide nanoparticles are combined with other intracellular proteins, both are subjected to the mechanical force of the EMF simultaneously [67]. In the presence of EMF, endogenous ferritin nanoparticles link with a camelid anti-GFP-transient receptor potential vanilloid 1 fusion protein, aGFP- TRPV1, causing calcium oscillations [63]. Unlike the non-thermal effect of EMF, EMF activates calcium channels through magneto-thermal interaction, when the EMF raises the temperature of magnetic nanoparticles, activating the temperature-sensitive TRV1 calcium channel and causing calcium ion inward flows [64]. An important mediator of the EMF-induced calcium ion oscillation is the superparamagnetic nanoparticles.

## Application of EMF in stem cells and its regulation of calcium ion pathway

### Bone repair effects of EMF

Extremely-low-frequency electromagnetic fields have been more widely recognized as a means to promote bone repair and have been validated several times in the treatment of animal fractures or bone defects, including the promotion of stem cell proliferation and osteogenic differentiation and angiogenic differentiation [68, 69]. Our research team [53] found that ELE-EMF can induce calcium oscillations in bone marrow stem cells, up-regulated calcium ion activates FAK pathway, cytoskeleton enhancement, and migration ability of stem cells in vitro is enhanced. On this basis, we grew rat stem cells on PCL/HA scaffolds, and the combined application of ELE-EMF and vascular endothelial growth factor activated β-catenin and promoted the osteogenic differentiation and angiogenesis of rat mesenchymal stem cells (MSCs) [16].

In the osteogenic differentiation of stem cells, the pERK and Wnt/β-catenin pathways play important roles, and calcium ion have a significant effect on both pathways in the differentiation regulated by electromagnetic fields [18, 70]. EMF can open volt-gated channels on stem cells and promote calcium ion inward flow [71, 72], thus promoting stem cells toward osteogenesis. However, the way in which calcium ion act on the Wnt/β-catenin pathway may be more complex, involving interactions between calcium ion and the cellular cilia system [73]. It has been shown [74] that the ability of EMF to activate the Wnt10b/β-catenin signaling pathway to promote osteogenic differentiation of cells depends on the functional integrity of primary cilia in osteoblasts. When primary cilia were inhibited using small interfering RNA (siRNA), the Wnt10b /β-catenin signaling pathway was no longer activated, and the ability of electromagnetic field to promote osteogenic differentiation was significantly diminished [75]. We suggest that after EMF activation of calcium channels, the interaction of calcium ion with cellular primary cilia further activates the Wnt/β-catenin pathway and promotes stem cells toward osteogenic differentiation, but the specific mechanisms involved need further investigation.

Nitric oxide (NO) plays an important role in the metabolic regulation as a star molecule [76]. Arthur [77] first documented the ability to produce NO after EMF stimulation of cells, by using a NO selective membrane electrode, and in further experiments used W-7, an inhibitor of CaM, which inhibited NO production. The production of NO is closely related to the intracellular calcium ion concentration and has a close relationship, as the calcium ion concentration increases, the cells produce NO [78]. NO produced by EMF, which also acts as a messenger molecule, further activates cGMP and PKG pathways, with a significant upregulation of ALP expression in cells and cellular differentiation toward osteogenesis [79]. EMF-mediated, calcium-dependent elevation of NO not only promotes osteogenic differentiation of stem cells, but also attenuates the inflammatory response and promotes wound healing and cartilage repair [80, 81].

### Cartilage repair by electromagnetic fields

The pro-differentiation and repair effects of EMF are not limited to contributing to bone differentiation; in different culture environments, stem cells exhibit the potential to differentiate into different cell types [19, 82]. In the chondrogenic environment, stem cells receive various physical stimuli and the synergistic effects of multiple ion channels and receptors promote the inward flow of calcium ion [83]. EMF may activate calcium channels other than voltage-gated calcium channels, and although there are relatively few reports of electromagnetic activation of purinergic receptors and TRP family of cation channels to further influence calcium oscillations, both may play an important role in EMF against inflammation and cartilage in osteoarthritis. Purinergic receptors play a key role in the development of cartilage as important molecular receptors that are widely present on the membranes of mesenchymal stem cells, chondrocytes, skeletal muscle cells, and other cells that receive purinergic signals [84]. Purinergic receptors can be divided into two major categories, P1 and P2 receptors, and it has been demonstrated that electromagnetic fields can upregulate the expression of Adenosine Receptors, with significant upregulation of A_2A_ and A_3_, exerting similar effects as adenosine receptor agonists, and can downregulate PGE(2) levels to achieve control of osteoarthritis [85, 86]. We found that EMF can upregulate P2X7 receptors in MSCs [40], and that the adenosine purinergic system acts as a classical regulatory pathway affecting the intracellular calcium ion homeostasis during the process of chondrogenic differentiation of cells [84].

It is the purinergic receptors and TRP family of cation channels which are sensitive to external mechanical or electrical signal stimuli that form the bridge between electromagnetic fields and changes in intracellular calcium ion concentration [87]. Purinergic receptors are functionally cation channels and are very sensitive to calcium ion that maintain intracellular calcium homeostasis within MSCs [84]. During cartilage formation, intracellular cascade reactions are sensitive to changes in calcium ion concentration [88]. In response to EMF stimulation, TGFβ expression is upregulated in stem cells, promoting stem cell differentiation toward chondrogenesis [89, 90]. In the process of chondrogenic differentiation, calcium ion have a crucial role in the activation of TGFβ [84, 91]. Although the relationship between the action of calcium ion and TGFβ under EMF conditions has not been reported, we speculate that TGFβ may be a downstream molecule of EMF-mediated calcium ion oscillation.

Cenk found that there is an orientation-dependent electromagnetic field for chondrogenic differentiation of stem cells, and in vitro cultured stem cells, electromagnetic field stimulation in the Z-axis direction is most favorable for the opening of TRPC1 receptors on stem cells and the inward flow of extracellular calcium ion. The inward flow of calcium ion promoted the phosphorylation of FAK, which further activated the ERK signaling pathway [20]. After this, intracellular expression of chondrogenic markers such as SOX9, COL2A1, and ACAN were significantly upregulated and cells differentiated toward chondrogenesis [92].

Calcium ion, as a second messenger to regulate physiological activities of cells, are closely related to mitochondria and ROS [93, 94]. Current studies suggest that excessive cellular reactive oxygen species (ROS), such as superoxide anion (O^2−^) and hydrogen peroxide (H_2_O_2_), can damage cells, causing varying degrees of inhibition of phosphatase expression in cascade reactions [95, 96], or more severely, leading to the destruction of mitochondria causing cell death [97], while, on the contrary, moderate amounts of ROS can regulate normal cellular physiological processes [98, 99]. Stimulation of stem cells by EMF generates a small amount of ROS to further activate EGFR signaling to induce differentiation of MSCs [100], while ROS produced by NADPH oxidase is indispensable in the differentiation process of primary chondrocytes [101]. Based on the current study, we hypothesize that the electromagnetic field-mediated calcium ion oscillations, which causes a small amount of ROS production in mitochondria, regulates the chondrogenic differentiation of cells, but further studies are needed to prove the exact mechanism.

## Electromagnetic field for tumor treatment

More and more studies have started to focus on the physiotherapy of tumors. Early knowledge of physiotherapy rested on the effects of thermal effects of physical stimuli on cells [102], but with the development and depth of research, it was gradually recognized that non-thermal effects can activate voltage-gated channels, a physiological process that plays an important role. Physical therapy can make cell membrane and mitochondrial membrane potential exhibit a hyperpolarized state, with a significant increase in intracellular calcium ion concentration and a concomitant increase in the concentration of reactive oxygen species and nitric oxide [103]. The imbalance of calcium ion homeostasis and disruption of mitochondria allow for an alteration of the tumor stem cells cycle, inhibiting cell proliferation and promoting apoptosis [104, 105]. EMF, as a widely noticed physical therapy, is able to modulate the voltage-gated channels of tumor stem cells through non-thermal effects, and the opening of CACNA1H channels allows a large amount of calcium ion inward flow, which eventually leads to the differentiation of HCC cells into quiescent cells with spindle-shaped morphology [22]. Meanwhile, RF-EMF was able to suppress tumor stem cells by activating the CAMKII/p38 MAPK signaling pathway after inducing calcium ion oscillation and by inhibiting the β-catenin/HMGA2 signaling pathway [21].

Interestingly, the effect of electromagnetic fields is not limited to tumor stem cells, but also inhibits the proliferation and development of tumor cells [106]. It has been shown that breast cancer cell lines exposed to ELE-EMF for 24 h showed a significant increase in intracellular ROS expression and an increased sensitivity to further radiotherapy [107]. Also breast cancer cell lines, after exposure to higher intensity EMF radiation, showed a significant increase in intracellular calcium ion and reactive oxygen species, which eventually led to necroptosis, while this programmed necrosis of tumor cells was able to be antagonized by the calcium blocker verapamil or the free radical scavenger n -acetylcysteine [23].

## Biosafety of electromagnetic fields

**Fig. 3:**
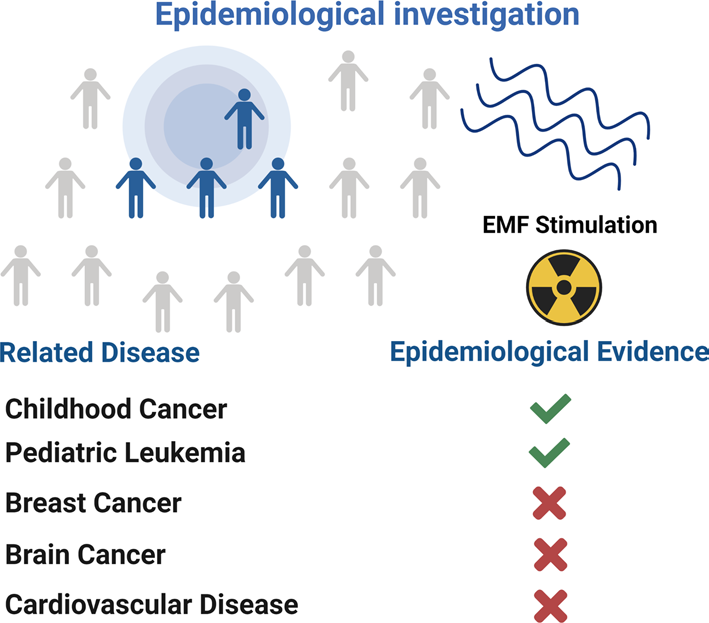
Epidemiological investigation results. According to epidemiological findings Electromagnetic fields may be a risk factor for cancer in children, but there is no evidence of cancer in adults. EMF is safe and reliable as a non-invasive treatment for adults. The figure was created with BioRender.com

The two-sided nature of the action of EMF inevitably raises concerns about their safety, and their therapeutic and tumor-causing effects have been controversial [108]. Some researchers have proposed the hypothesis that EMFs cause an imbalance in ion homeostasis, leading to local inflammation or tumorigenesis [38], which means that very low frequency or radiofrequency EMFs can exert biological effects while irregularly opening multiple ion channels (Na^+^, K^+^ and Ca^2+^) [109–111], causing an imbalance in intracellular ion homeostasis and producing excessive OS/ROS leading to DNA damage [112, 113], but this postulation lacks direct experimental evidence and necessary epidemiological investigations. For normal animals, exposure of animals to 1.5 mT at 50 Hz intensity had no significant adverse effects [114]. In nearly 50 years of research, only low-frequency EMF has been confirmed as a risk factor for pediatric leukemia, and epidemiological studies have shown that ELE-EMF is not a risk factor for breast cancer or cardiovascular disease, and according to the current studies, there is not enough evidence to show that electromagnetic fields pose a health threat to adults [31, 115–117] (Fig. 3).

High-frequency electromagnetic waves are closely related to modern communication in daily life, and therefore research related to high-frequency electromagnetic fields and human health has been ongoing [118, 119]. Although it has been suggested that when the frequency of EMF reaches 1800 MHz, this high-energy EMF radiation causes chromosomal alterations in the nucleus of cells [120], its cytotoxicity and gene damage toxicity are harmful [121], potentially increasing the risk of reproductive cancers [122]. However, there is not enough evidence in epidemiological statistics to be able to suggest that high-frequency EMFs are a high-risk factor for tumorigenesis [123].

It has been shown that electromagnetic fields promote apoptosis in tumor cells such as B16-BL6 mouse melanoma cells, MDA-MB-231, MDA-MB-468, BT-20, and MCF-7 human breast and HeLa cervical cancer cells, but do not affect non-malignant cells [124]. Tumor cells have a large number of variants in their calcium channels compared to normal cells, and these variants lead to abnormal biological behavior of tumor cells [15]. Electromagnetic fields are able to open voltage-gated channels in the cell membrane, causing an imbalance of calcium ion, which affects tumor proliferation and promotes apoptosis [105, 125]. This radiofrequency EMF has high energy and certain penetration, and the therapeutic principle of radiofrequency EMF is to cause excessive or irregular opening of ion channels thus causing ion imbalance and excessive production of ROS which in turn leads to DNA damage, and the question that remains is whether this mechanism acts on normal cells.

## Discussion

**Fig. 4:**
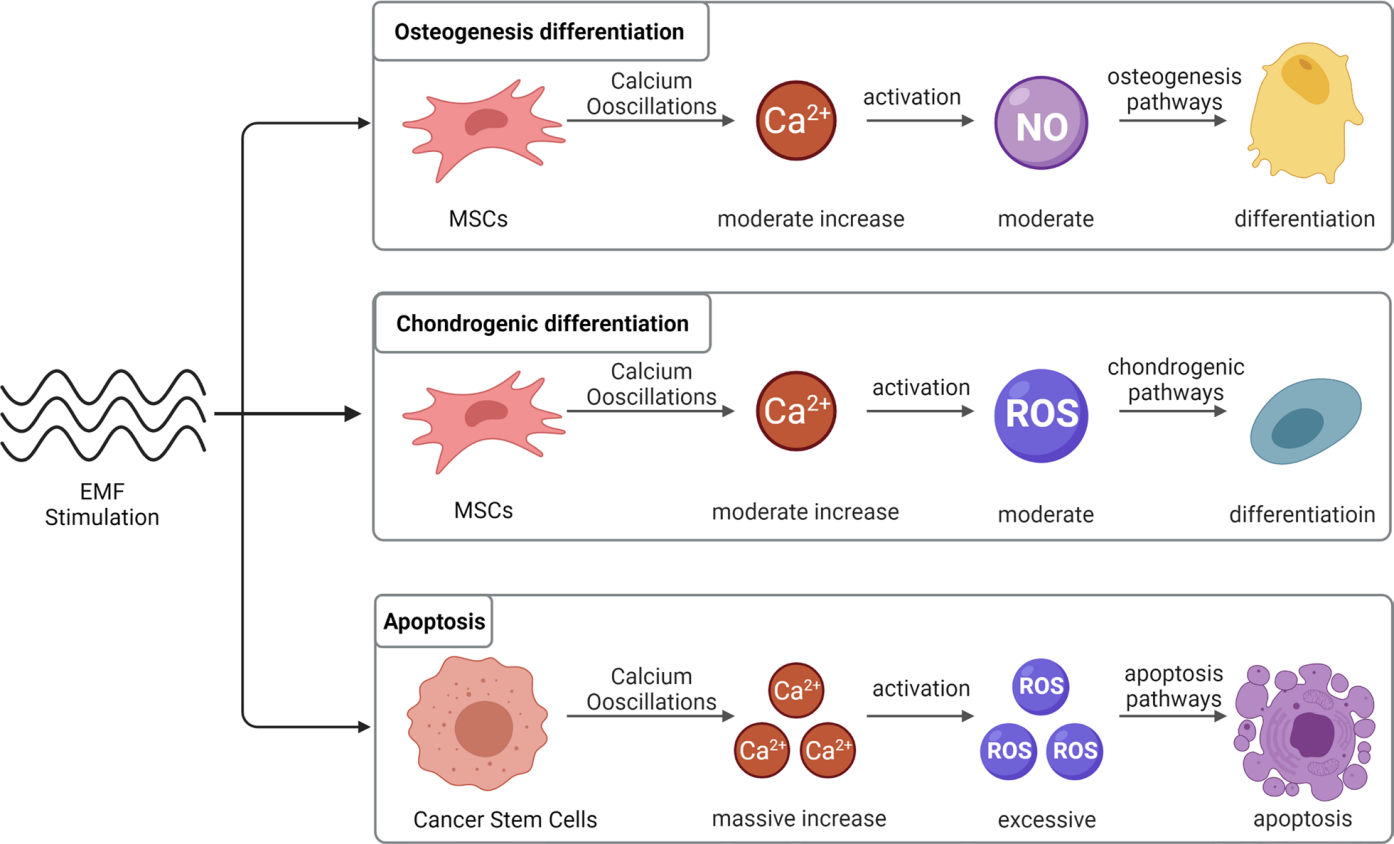
Electromagnetic fields-dependent calcium-mediated stem cell and tumor cell fate. EMF mediates calcium ion oscillation in stem cells, and calcium ion activate NO molecules, which further activate the osteogenic differentiation pathway and promote osteogenic differentiation of stem cells; calcium ion can activate a small amount of ROS molecules, which further activate the chondrogenic differentiation pathway and promote chondrogenic differentiation of stem cells. EMF causes calcium overload in tumor cells and a large amount of ROS activation, leading to apoptosis. The figure was created with BioRender.com

As research continues, EMF combined with stem cells have also been shown to be used in the treatment of osteoporosis [126], soft tissue injuries such as rotator cuff [127] and inhibition of tumor development [128]. At the same time, the relationship between EMF and tumors has become a controversial topic [119], but there is little epidemiological basis for the carcinogenicity of EMF, in contrast to sophisticatedly designed experiments demonstrating the pro-apoptotic ability of EMF on tumor stem cells [129].We believe that the two-sided ability of EMF to promote stem cells differentiation and tumor stem cells apoptosis is due to three reasons: first, EMF as an energy field promotes the opening of ion channels and the inward flow of calcium ion when we use lower frequencies and appropriate intensities [20, 53, 54], and causes calcium overload when the electromagnetic field intensity or frequency is further increased [21–24], second, EMF can regulate multiple ions in cells, and calcium ion play a key role [92, 130], calcium ion acts as a second messenger that can activate downstream molecules such as NO, ROS [77, 100, 102, 106], which further regulate cell differentiation or apoptosis through the β-catenin pathway; thirdly, the variation of calcium channels in tumor stem cells themselves, which makes the regulation of EMF polarized [131] (Fig. 4).

Specifically, this difference in biological effects is closely related to the electromagnetic field parameters, the cell culture environment and the type of activated ion channels. When low or extremely low frequency EMF is selected, i.e. frequencies between 0 and 75 Hz and intensities between 0 and 1 mT, EMF is able to exert osteogenic or chondrogenic effects. In this case, a corresponding cell culture environment is also required. Furtherly, the EMF activates different types of calcium channels. In the osteogenic environment, EMF primarily activates voltage-gated calcium channels to promote osteogenic differentiation. Besides, in the chondrogenic environment, EMF mainly activates receptor-like cation channels, such as purinergic receptors or several members of the transient receptor potential (TRP) channel family [84–86]. In general, after the activation of these ion channels, intracellular calcium ion concentrations will rise and downstream proteins of multiple cascade reactions regulated [84]. RF EMF with frequencies of 27.12 MHz and 835 MHz have a therapeutic biological effect on tumors. The overexpressed calcium channels in tumor cells can be hyperactivated by these high energy EMF, thus resulting an imbalance in calcium homeostasis, which then causes a DNA damage through ROS overexpression and an ultimate apoptosis [21, 32, 129]. However, it is noted that low-frequency EMF should not be used among children, because EMF can lead to the development of pediatric leukemia [31]. Meanwhile, we need to prevent damage to surrounding tissues from RF EMF due to its excessive energy [115].

The biological effects of EMF are mainly mediated through calcium channels, but in the cell membrane, there are multiple voltage-gated types of ion channels (Na^+^, K^+^ and Ca^2+^, etc.), and EMF have a modulatory effect on these ion channels, therefore, the possibility of EMF mediating biochemical reactions through these ion channels has attracted interest [38]. Under the induction of ELF-EMF, researchers have successfully recorded the alteration of Na^+^ and K^+^ currents in cell membranes using the cell membrane clamp technique [111]. EMF have now been found to affect a variety of potassium channels (A-type K^+^, delayed rectifier K^+^, M-type K^+^, fast-inactivating transient (IK, A), and dominant-sustained (IK, V) channels) [132]. EMF-mediated alterations in potassium currents may cause physiological and pathological changes in cellular metabolism, immune regulation, inflammation, or tumors, but further mechanisms are unclear.

## Conclusion

In this paper, we summarize the applications of EMF in combination with stem cells based on existing studies and point out that EMF with different parameters in different cell culture environments can mediate stem cells towards different cell fates, osteogenic differentiation, chondrogenic differentiation or apoptosis. We suggest that EMF induces calcium oscillations through non-thermal effects directly on superparamagnetic nanoparticles, and in MSCs, upregulation of calcium ion regulates proliferation and differentiation. In contrast, in tumor stem cells, excessive activation of calcium ion channels by EMF leads to drastic changes in calcium homeostasis, which eventually leads to apoptosis. We further summarize the downstream molecules of EMF-induced calcium oscillations and elucidate their biosafety. We hope that this review will provide hints for further studies on EMF, stem cells and calcium ion to find more efficient superparamagnetic nanoparticles that can better integrate EMF with tissue engineering and more effectively solve clinical problems related to bone repair, cartilage repair and bone tumors.

## Data Availability

Not applicable.

## Abbreviations

EMF: Electromagnetic fields
ELE-EMF: Extremely low frequency electromagnetic fields
RF-EMF: Radio frequency electromagnetic fields
PEMF: Pulsed electromagnetic fields
MSCs: Mesenchymal stem cells
BMSCs: Bone marrow mesenchymal stem cells
ADSCs: Adipose Derived Stem Cells
HCC: Hepatocellular carcinoma
ROS: Reactive oxygen species
NO: Nitric oxide
ALP: Alkaline phosphatase
CaM: Calmodulin
ATP: Adenosine triphosphate
VGCC: Voltage-Gated Calcium Channel
VEGF: Vascular endothelial growth factor
PCL: Polycaprolactone
CMC: Carboxymethyl cellulose
HA: Hydroxyapatite
